# The Role of *Cannabis sativa* L. as a Source of Cannabinoids against Coronavirus 2 (SARS-CoV-2): An In Silico Study to Evaluate Their Activities and ADMET Properties

**DOI:** 10.3390/molecules27092797

**Published:** 2022-04-27

**Authors:** Ahmed E. Altyar, Fadia S. Youssef, Maram M. Kurdi, Renad J. Bifari, Mohamed L. Ashour

**Affiliations:** 1 Department of Pharmacy Practice, Faculty of Pharmacy, King Abdulaziz University, P.O. Box 80260, Jeddah 21589, Saudi Arabia; aealtyar@kau.edu.sa; 2 Department of Pharmacognosy, Faculty of Pharmacy, Ain-Shams University, Abbasia, Cairo 11566, Egypt; fadiayoussef@pharma.asu.edu.eg; 3 Faculty of Pharmacy, King Abdulaziz University, P.O. Box 80260, Jeddah 21589, Saudi Arabia; mkurdi0012@stu.kau.edu.sa (M.M.K.); rbefari0001@stu.kau.edu.sa (R.J.B.); 4 Department of Pharmaceutical Sciences, Pharmacy Program, Batterjee Medical College, P.O. Box 6231, Jeddah 21442, Saudi Arabia

**Keywords:** *Cannabis sativa*, cannabinoids, COVID-19, molecular docking, ADME/TOPKAT prediction, chemometric analysis

## Abstract

*Cannabis sativa* L. is an annual herbaceous plant that belongs to the family Cannabinaceae. In this study, the potential use of forty-five cannabinoids, previously identified from *Cannabis sativa* to alleviate COVID-19 infection via prohibition of crucial SARS-CoV-2 proteins using molecular docking, was examined. In silico studies were performed on three vital enzymes that serve as principle therapeutic targets to prevent SARS-CoV-2 replication. These enzymes are the main protease SARS-CoV-2 M^Pro^, papain-like protease SARS-CoV-2 PL^pro^ and angiotensin-converting enzyme 2 (ACE2). Regarding SARS-CoV-2 M^Pro^, cannabichromanon (**32**) showed the best fitting within its active centers, followed by cannabinolic acid (**22**) and cannabinol (**21**), displaying ∆G of −33.63, −23.24, and −21.60 kcal/mol, respectively. Concerning SARS-CoV-2 PL^pro^, cannabichromanon (**32**) followed by cannabinolic acid (**22**) and cannabicyclolic acid (**41**) revealed the best binding within its active pockets owing to multiple bond formation with ∆G values of −28.36, −22.81, and −19.89 kcal/mol. Furthermore, cannabichromanon (**32**), cannabinolic acid (**22**), and cannabinol (**21**) showed considerable fitting within the active sites of angiotensin-converting enzyme 2 (ACE2) evidenced by their significant ∆G values that were estimated as −41.77, −31.34, and −30.36 kcal/mol, respectively. ADME/TOPKAT (absorption, distribution, metabolism, excretion, and toxicity) evaluation was performed on the tested cannabinoids to further explore their pharmacokinetics, pharmacodynamics, and toxicity properties. The results indicated the considerable pharmacokinetic, pharmacodynamic, and toxicity properties of cannabinol (**21**), cannabinolic acid (**22**), cannabichromanon (**32**), and cannabicyclolic acid (**41**) that showed best fitting scores within the active sites of the tested enzymes. Multivariate data analysis revealed that cannabichromanon and cannabinolic acid showed a discriminant nature and hence can be incorporated in pharmaceutical dosage forms to alleviate COVID-19 infection.

## 1. Introduction

*Cannabis sativa* L. is an annual herbaceous plant originally cultivated in central Asia. It is also named Indian hemp, whereas marijuana is a Mexican term that has recently been ascribed to the dried flowers and leaves of the cannabis plant; its Arabic name hashish refers to the plant resin gum [1]. Traditionally, it has been employed as a food source, and has been utilized for the provision of oil, medicine, and fibers alongside its popularity for religious, recreational, and therapeutic purposes [1,2].

*C. sativa* belongs to the family Cannabinaceae and is a dioecious annual plant but is also rarely monoecious. The stems, bracts, and leaves of *C. sativa* are covered with glandular epidermal trichomes, containing the chief phytoconstituents, particularly phytocannabinoids and terpenoids. The former constitute the defense of the plant versus pests and herbivores, whereas the latter is responsible for its typical odor [3].

Cannabinoids, a class of terpene phenolic components, represent the major class of active metabolites existing in *C. sativa* which are concentrated chiefly within the female flowers in the trichome cavity [4]. Among cannabinoids, Δ-9-tetrahydrocannabinol represents the main psychoactive compound; cannabidiol (CBD) constitutes the principle non-psychotic active compound [3]. Furthermore, *Cannabis sativa* has been reported to possess various therapeutic activities that are mainly attributed to the presence of cannabinoids. These medicinal properties hinder the progression of neurodegenerative disorders, prohibit breast cancer cell proliferation, and include the ability to alleviate inflammation, chronic pain, multiple sclerosis, epilepsy, glaucoma, and nausea [5,6].

Coronavirus disease 2019 (COVID-19) is triggered by a type of transmissible pathogenic human severe acute respiratory syndrome coronavirus 2 (SARS-CoV-2) which belongs to the *Beta* coronavirus type [7]. SARS-CoV-2 infection is characterized by severe respiratory distress evidenced by shortness of breath, dry cough, and fever, resulting in high morbidity and mortality [8]. No specific therapeutic strategy is available to treat COVID-19 infection; only vaccination is available that can decrease the severity of the health disorders caused by the infection. Thus, exploring effective treatments, particularly of natural origin and with less severe adverse effects, is thought to be essential worldwide [9].

Molecular docking performed in silico is an enhanced technique that can enable the discovery of natural secondary metabolites revealing considerable activity. It is performed based on knowledge of the different chemical structures which exist gathered from well-known databases and aims to predict the bioactivity of certain entities with respect to specific target molecules. Via virtual screening of molecular docking, it is possible to reduce the time, effort, and resources which are required to perform in vitro and in vivo studies on isolated compounds. Furthermore, by identification of inactive compounds, the total number of compounds for further investigation can be reduced [10,11].

This study aimed to explore the potential use of popular cannabinoids to alleviate COVID-19 infection via the prohibition of crucial SARS-CoV-2 proteins using molecular docking. In silico studies were performed on three vital enzymes that serve as principle therapeutic targets to prevent SARS-CoV-2 replication. These enzymes were the main protease SARS-CoV-2 M^Pro^, papain-like protease SARS-CoV-2 PL^pro,^ and angiotensin-converting enzyme 2 (ACE2). In addition, ADME/TOPKAT (absorption, distribution, metabolism, excretion, and toxicity) evaluation was performed on the tested cannabinoids to further explore their pharmacokinetic, pharmacodynamic, and toxicity properties. Multivariate data analysis was performed using principal component analysis (PCA) as a means of unsupervised pattern recognition to better visualize the differences among the tested compounds.

## 2. Results

### 2.1. Selection of Secondary Metabolites

**Figure 1:**
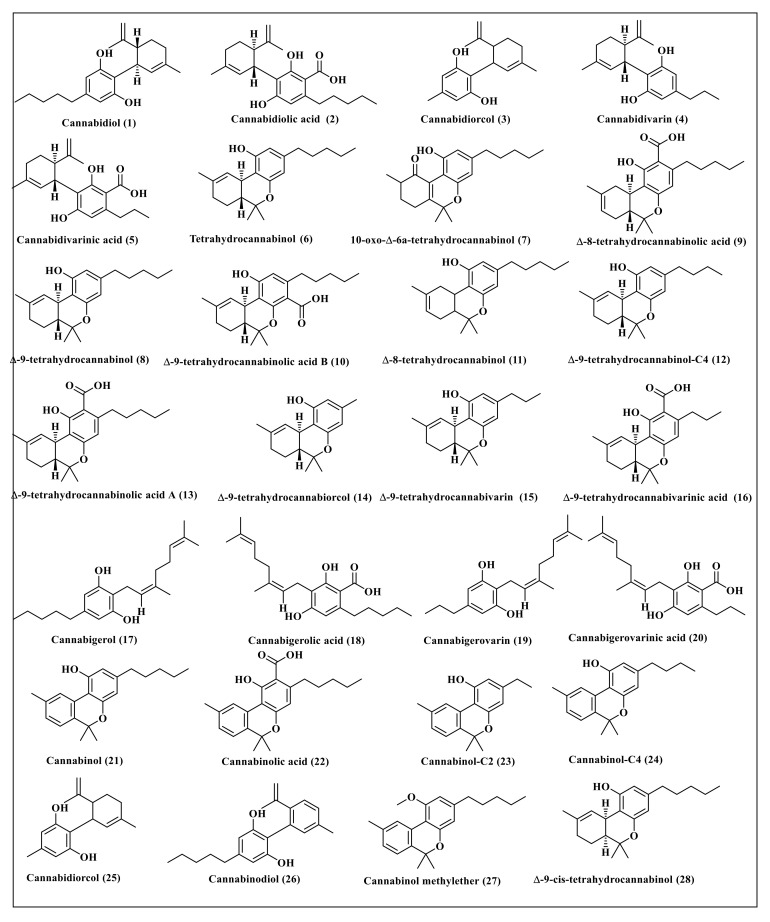
The chemical structures of some of the major cannabinoids present in *Cannabis sativa*.

**Figure 2:**
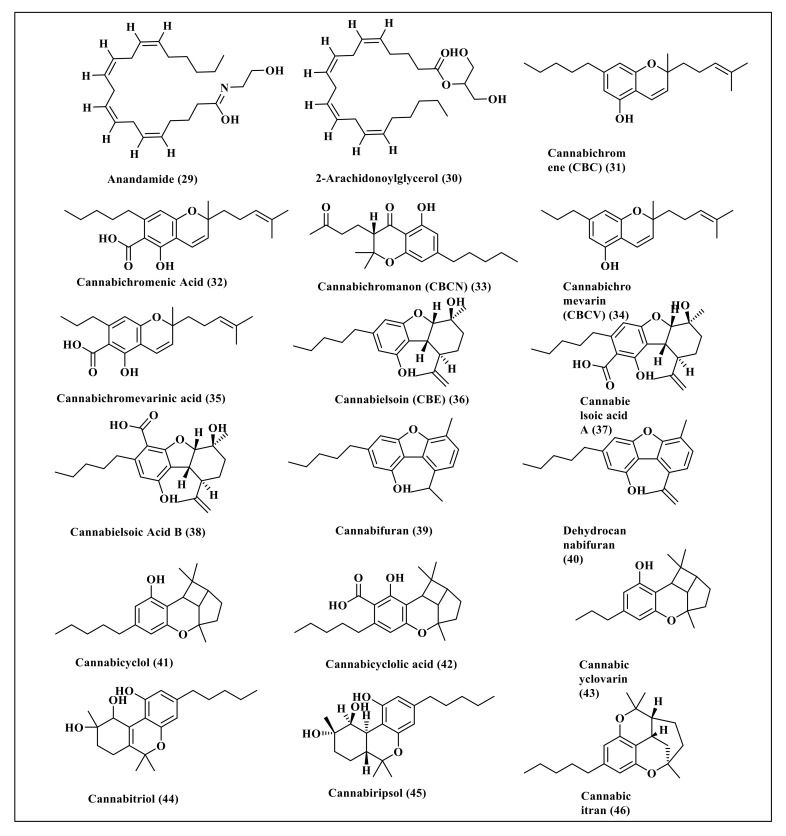
The chemical structures of some of the primary endocannabinoids and minor cannabinoids present in *Cannabis sativa*.

*Cannabis sativa* L. is a rich source of cannabinoids, some of which represent well-known classes and thus were selected for this study. These secondary metabolites include cannabidiol (**1**) and its derivatives, cannabidiolic acid (**2**), cannabidiorcol (**3**), cannabidivarin (**4**), and cannabidivarinic acid (**5**) in addition to tetrahydrocannabinol (**6**) and its derivatives comprising 10-oxo-delta-6a-tetrahydrocannabinol (**7**), ∆-9-tetrahydrocannabinol (**8**), ∆-8-tetrahydrocannabinolic acid (**9**), ∆-9-tetrahydrocannabinolic acid B (**10**), ∆-8-tetrahydrocannabinol (**11**), ∆-9-tetrahydrocannabinol-C4 (**12**), ∆-9-tetrahydrocannabinolic acid A (**13**), ∆-9-tetrahydrocannabiorcol (**14**), ∆-9-tetrahydrocannabivarin (**15**) and ∆-9-tetrahydrocannabivarinic acid (**16**). In addition, cannabigerol (**17**) and its derivatives, represented by cannabigerolic acid (**18**), cannabigerovarin (**19**), cannabigerovarinic acid (**20**), together with cannabinol (**21**) and its derivatives, cannabinolic acid (**22**), cannabinol-C2 (**23**), cannabinol-C4 (**24**), cannabinodiol (**25**), cannabinol methyl ether (**26**), and ∆-9-cis-tetrahydrocannabinol (**27**) were also studied. Primary endocannabinoids, such as anandamide (**28**) and 2-arachidonoylglycerol (**29**), were also included in the study. Furthermore, some familiar minor cannabinoids, such as cannabichromene (**30**), cannabichromenic acid (**31**), cannabichromanon (**32**), cannabichromenevarin (**33**), cannabichromevarinic acid (**34**), cannabielsoin (**35**), cannabielsoic acid A (**36**), cannabielsoic acid B (**37**), cannabifuran (**38**), dehydrocannabifuran (**39**), cannabicyclol (**40**), cannabicyclolic acid (**41**), cannabicyclovarin (**42**), cannabitriol (**43**), cannabiripsol (**44**) and cannabicitran (**45**) were also included in the study. The chemical structures of the studied cannabinoids are represented in Figure 1 and Figure 2.

### 2.2. Molecular Docking Studies

**Figure 3:**
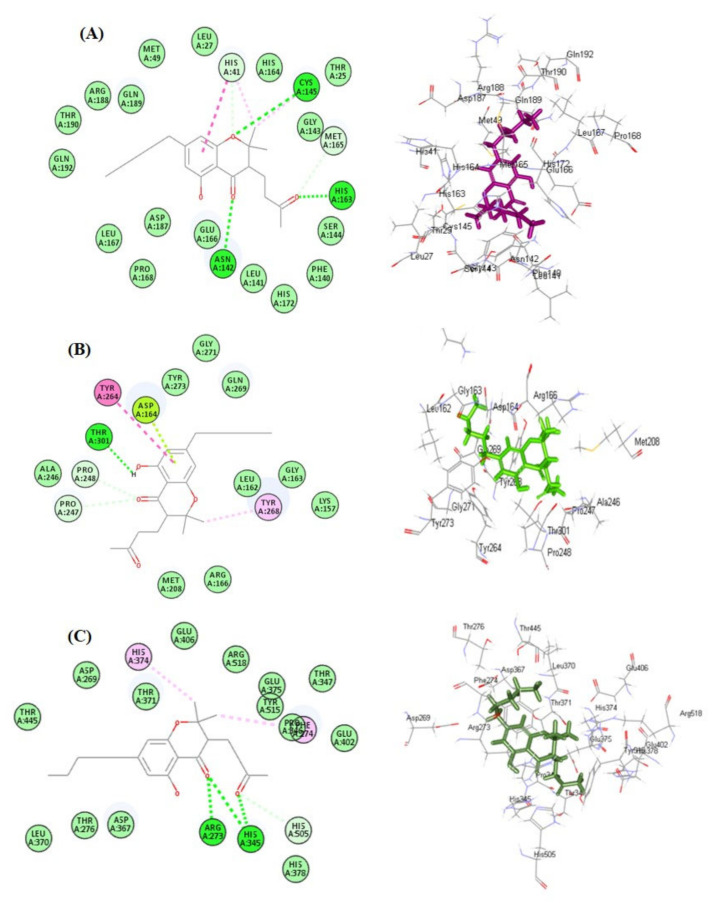
2D and 3D binding of cannabichromanon within the active sites of SARS-CoV-2 M^Pro^ (**A**); SARS-CoV-2 PL^pro^ (**B**) and ACE2 (**C**).

Results of molecular docking revealed that cannabinoids showed variable degrees of interaction with the enzymes, as illustrated in Table 1. Molecular docking was performed using a pH-based ionization mode that mimics the physiological conditions that occur within the human body. For each compound, the ten most favorable docking poses were selected by the software; in Table 1 the best docking pose for each compound is displayed as revealed by the software. Regarding SARS-CoV-2 M^Pro^, cannabichromanon (**32**) showed the best fitting within its active centers, followed by cannabinolic acid (**22**) and cannabinol (**21**), displaying ∆G of −33.63, −23.24, and −21.60 kcal/mol, respectively. These compounds showed superior activity in comparison to the SARS-CoV-2 M^Pro^ co-crystalized ligand (FHR/PRD_002347), which displayed ΔG of −4.58 kcal/mol. The firm binding of these compounds with the binding sites may be attributed to the formation of different types of bond between the functional groups existing in the bioactive compounds and the amino acid moieties existing at the active sites. Cannabichromanon (**32**) formed three conventional H-bonds with Cys145, His163, Asn142; one π-π T-shaped bond with His41; two C-H bonds with His41 and Met165; one π-alkyl bond with His41 and one alkyl bond with Cys145 (Figure 3A). Cannabinolic acid (**22**) showed one conventional H-bond with His164, one C-H bond with Met165, two π-alkyl bonds with His163, and Met165, one alkyl bond with Cys145, in addition to one π-sulfur bond with Cys145 (Supplementary Materials Figure S1). However, cannabinol (**21**) displayed one conventional H-bond with Glu166, one C-H bond with Cys145, three π-alkyl bonds with His163, His41, and Met165; one alkyl bond with Cys145 where the alkyl bond was used to describe the attractive stabilizing interactions between two alkyl moieties involving amino acid side-chain residues and bound ligands (Figure S1).

Concerning SARS-CoV-2 PL^pro^, cannabichromanon (**32**), followed by cannabinolic acid (**22**) and cannabicyclolic acid (**41**), revealed the best binding within its active pockets, owing to multiple bond formation with ∆G values -28.36, −22.81, and −19.89 Kcal/mol. These compounds showed higher activity when compared to the SARS-CoV-2 PL^pro^ co-crystalized ligand (Y97) that displayed ∆G of −4.08 kcal/mol. Cannabichromanon (**32**) formed one H-bond with Thr301; two C-H bonds with Pro247 and Pro248; one π-π T-shaped bond with Tyr264; one alkyl bond with Tyr268, in addition to one π lone-pair bond with Asp164 existing at the binding site (Figure 3B). Cannabinolic acid (**22**) formed one H-bond with Asp164; one π-π T-shaped bond with Tyr268; one π-alkyl bond with Tyr268, in addition to the formation of one alkyl bond with Pro248 (Figure S2). Similarly, cannabicyclolic acid (**41**) formed one H-bond with Asp164, one π-π T-shaped bond with Tyr268, and four π-alkyl bonds with Pro247 Pro248, and Tyr268 (Figure S2).

Furthermore, cannabichromanon (**32**), cannabinolic acid (**22**), and cannabinol (**21**) showed considerable fitting within the active sites of ACE-2 evidenced by their significant ∆G values that were estimated as −41.77, −31.34, and −30.36 kcal/mol, respectively, compared to the ACE2 co-crystalized ligand (XX5) that revealed ∆G of −72.19 kcal/mol. Cannabichromanon formed three H-bonds with Arg273 and His345; one C-H bond with His505; two π-alkyl bonds with His374 and Phe274 (Figure 3C). However, cannabinolic acid showed significant binding via the formation of three conventional H-bonds with Pro346, His345, Arg518; one π-π stacked bond with Phe274; two π-alkyl bonds with Phe274, in addition to one π-cation bond with Arg273 (Figure S3). However, cannabinol formed one H-bond with Arg518; one π-π T-shaped bond with Phe274; one π-cation bond with Arg273; two C-H bonds with His374 and Pro346, in addition to three π-alkyl bonds with Phe274, Pro346 and His345 (Figure S3). The presence of cannabichromanon (**32**) inside the binding pockets of SARS-CoV-2 M^Pro^ (**A**), SARS-CoV-2 PL^pro^ (**B**), and ACE-2 (**C**) displaying areas of aromaticity hydrogen bond formation with receptor donors are colored green in the figure, whereas the receptor acceptors take the cyan color, hydrophobicity regions range from brown for hydrophobic to blue for hydrophilic, and solvent accessibility (SAS) of the receptor residues range from blue, expressing the exposed regions, to green for the buried areas (Figure S4). The 2D binding modes of the respective co-crystalized ligands of SARS-CoV-2 M^Pro^ (A), SARS-CoV-2 PL^pro^ (B), and ACE-2 (C) within the active sites are displayed in Figure S5.

The bioactive compounds, particularly cannabichromanon, showed nearly similar activity when compared to remdesivir, the standard antiviral drug, with respect to SARS-CoV-2 M^Pro^ and ACE2, where remdesivir showed ∆G values of −35.56 and −44.62 kcal/mol, respectively. However, cannabichromanon showed superior fitting in SARS-CoV-2 PL^pro^ when compared to remdesivir (∆G = 2.28 kcal/mol). Remdesivir formed six H-bonds with Phe140, His163, Gly143, Glu166, Cys145, Gln189; one π-π bond His41; and one π-sulphur bond and one alkyl bond with Met165, in addition to two C-H bonds with His41 and Asn142 within the active site of SARS-CoV-2 M^Pro^. It formed seven H-bonds with Glu402, His374, Arg273, Tyr515, Tyr510 and His315; two π-π bonds His374 and Tyr510; one π-cation bond Arg518; and six C-H bonds with His378, Ala340, Glu402, Glu375, His315 and Pro346 in the ACE-2 active site (Figure S6).

### 2.3. ADME/TOPAKT Evaluation

**Figure 4:**
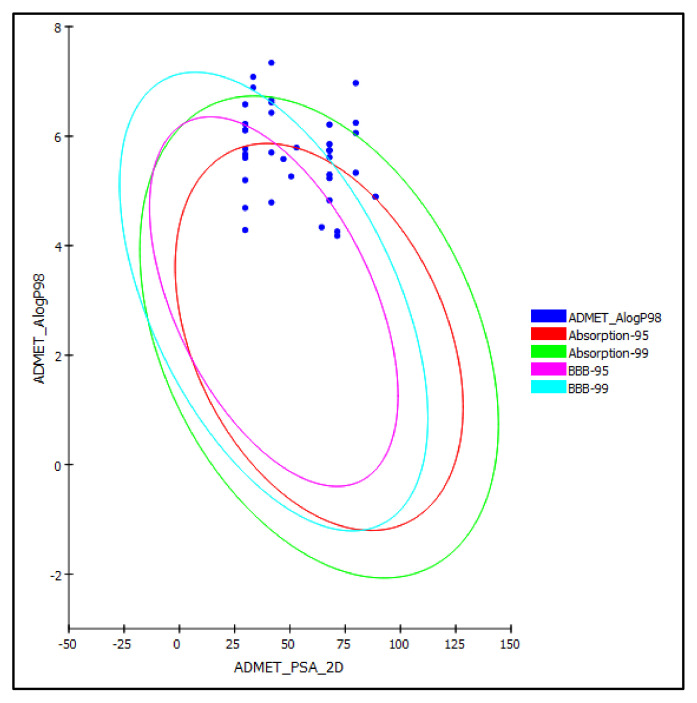
ADMET plot for selected cannabinoids present in *C. sativa* displaying 95% and 99% confidence limit ellipses corresponding to the blood-brain barrier (BBB) and the human intestinal absorption models in ADMET_AlogP98.

The selected cannabinoids were subjected to ADME/TOPAKT prediction to determine their pharmacodynamic, pharmacokinetic, and toxicity properties. Regarding *ADME* prediction, the results illustrated in Table 2 indicate that most of the tested cannabinoids showed good and moderate human intestinal absorption lying inside the 99% absorption ellipse, as illustrated in the ADMET plot (Figure 4). In contrast to cannabidiolic acid (**2**), cannabigerol (**17**), cannabigerolic acid (**18**), cannabifuran (**38**), and dehydrocannabifuran (**39**) showed low absorption lying outside the 99% absorption ellipse. The examined compounds showed either very high or high penetration via BBB taking 0 and 1 values, respectively, lying within the 99% confidence eclipse of BBB, or undefined penetration via BBB with a value of 4 lying outside the 99% confidence eclipse of BBB, as illustrated in Table 2 and Figure 4. The tested cannabinoids revealed very low and low solubility limits taking values of 1 and 2, respectively. For plasma protein binding, all the examined compounds showed more than 90% binding, except for 2-arachidonoylglycerol (**29**), which showed less than 90% binding. Most of the compounds showed no inhibition to CPY2D6, except cannabidiol (**1**), 10-oxo-∆-6a-tetrahydrocannabinol (**7**), cannabigerol (**17**), cannabigerovarin (**19**), and cannabigerovarinic acid (**20**); the compounds varied in their degree of hepatotoxicity, as shown in Table 2. 

For the TOPKAT examination, the results displayed in Table 3 revealed that all the compounds were found to be non-mutagenic in the Ames test. In addition, most of the compounds were shown to be non-carcinogenic towards male and female rats FDA, except for cannabichromenevarin (**33**), cannabicyclovarin (**42**), and cannabitriol (**43**) that showed some carcinogenicity towards female rats FDA. In addition, the selected cannabinoids showed rat oral LD50 values ranged between 0.09–5.32 g/kg body wt. Similarly, they revealed rat chronic LOAEL (lowest observed adverse effect level) values of 0.02–0.42 g/kg body wt. From Table 3, it is evident that most of the compounds were non-irritant to the skin. However, they showed severe irritation to the eye. ADME/TOPAKT analyses reflected the considerable pharmacokinetic, pharmacodynamic, and toxicity properties of cannabinol **(21)**, cannabinolic acid (**22**), cannabichromanon (**32**), and cannabicyclolic acid (**41**) that showed best fitting scores within the active sites of the tested enzymes and hence can be incorporated in pharmaceutical dosage forms to alleviate COVID-19 infection.

### 2.4. Chemometric Analysis

**Figure 5:**
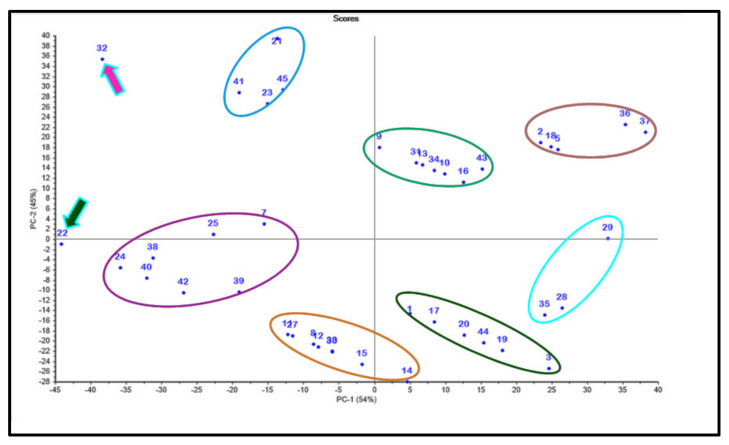
PCA score plot of different cannabinoids using a chemometric unsupervised pattern recognition technique; compounds are given numbers as listed in Table 1.

Data obtained from the binding free energies of the selected cannabinoids versus the three tested enzymes and the values obtained from ADMET evaluation were subjected to multivariate data analysis. The results illustrated in Figure 5 showed the clustering of the examined compounds into seven clusters that reflected the similarity of the clustered compounds in their chemical structures that, in turn, were related to their biological, pharmacokinetic, and pharmacodynamic properties. The PCA score plot illustrated in Figure 5 displays the principal components (PCs) PC1 and PC2 that accounted for 54% and 45% of the total variance, respectively, reflecting their significant potential to discriminate among the tested cannabinoids. Both PCs effectively discriminated among all the compounds, classifying them into seven clusters; however, the compounds cannabinolic acid (**22**) and cannabichromanon (**32**) displayed unique properties and thus were not incorporated in any of the clusters. Cannabichromanon (**32**) lay in the upper left quadrant showing negative values for PC1 and positive values for PC2; however, cannabinolic acid (**22**) lay in the lower left quadrant showing negative values for both PC1 and PC2. This pattern highlights the discriminant nature of these compounds, where they showed best fitting within all examined enzymes’ active sites and acceptable pharmacodynamic and pharmacokinetic behavior.

## 3. Discussion

It has long been known that viral infections are difficult to treat and are resistant to chemotherapy. This difficulty is due to the closeness between the viral replicative cycle and that of the normal cell, making the suppression of viral reproduction hazardous to normal cell division. However, via appropriate elucidation of virus-specific steps during their replication, it becomes possible to discover the main sites that chemotherapeutic antiviral agents could target [12]. 

The SARS-CoV-2 genome encodes about 25 proteins that are crucial for the virus to trigger human infection. The spike (S) protein is responsible for the initiation of infection by the virus after recognition and binding with angiotensin-converting enzyme-2 in human lung cells. The viral and human proteins are cleaved by two proteases. Meanwhile, RNA polymerase is responsible for the synthesis of viral RNA. SARS-CoV-2 MPro (main protease) performs a vital role during the lifecycle of SARS-CoV-2 within the human body. Both SARS-CoV-2 papain-like protease and SARS-CoV-2 Mpro cause the translation of viral RNA polyproteins. SARS-CoV-2 Mpro acts considerably at 11 cleavage sites of Leu-Gln ↓ (Ser, Ala, Gly) of the polyprotein replicase 1ab. Mpro is present among coronaviruses, with various Mpro substrates in various coronaviruses possessing common features. SARS-CoV-2 Mpro forms 12 non-structural proteins, including Nsp4 and Nsp16, via the cleavage of viral polyproteins, comprising RNA-dependent RNA polymerase (RdRp, Nsp12), as well as the helicase Nsp13 [13,14,15]. COVID-19 infection depends upon host cell factors, such as angiotensin-converting enzyme 2 (ACE2). The entrance of coronaviruses to the host cell is accommodated by the tight binding of the virus spike (S) proteins to the cell receptors that enhance viral entrance and adherence to the cell surface that subsequently results in the infection. SARS-S engages angiotensin-converting enzyme 2 (ACE2); the ability of the virus to bind to ACE2 was found to be the main cause of SARS-CoV transmissibility. Thus the inhibitory effect of the ACE2 catalytic pocket by effective metabolites could change the conformation of ACE2, and inhibit SARS-CoV-2 entrance in the host cells via ACE2 [8,16]. Thus, the main protease SARS-CoV-2 M^Pro^, papain-like protease SARS-CoV-2 PL^pro,^ and angiotensin-converting enzyme 2 (ACE2) serve as the best therapeutic targets to prevent coronavirus replication

It is worth highlighting that in vitro assay for the inhibition of COVID-19 infections is very expensive and requires extensive precautions. So, it is better to test compounds that are expected to show significant activity. This has recently been achieved via computational chemistry using virtual screening. Computational analyses speed up these approaches and enable the simultaneous handling of millions of pieces of data [17]. The main target of virtual screening is reduction in the time, resources, and effort necessary for both the in vitro and in vivo screening of defined compounds. Thus, the total number of compounds for further processing can be substantially reduced by the initial prediction of inactive compounds. Consequently, the hit scores in both in vitro and in vivo assays are dramatically increased by excluding inactive compounds compared to random assessment without preliminary virtual screening [18,19]. 

Many researchers have adopted molecular modeling to screen the possibility of using active constituents derived from natural products to combat COVID-19 infection. A recent in silico study was conducted on certain cannabinoids using the SARS-CoV-2 Mpro enzyme as the main target, followed by in vitro antiviral activity versus SARS-CoV-2. Δ9–tetrahydrocannabinol and cannabidiol showed the most significant antiviral potential displaying IC_50_ of 10.25 and 7.91 μM, respectively, which was comparable to lopinavir, chloroquine, and remdesivir with IC_50_ ranging between 8.16–13.15 μM. In the present study, the forty-five selected cannabinoids were tested in silico within the binding pockets of the main protease SARS-CoV-2 M^Pro^, papain-like protease SARS-CoV-2 PL^pro,^ and angiotensin-converting enzyme 2 (ACE2). With respect to SARS-CoV-2 M^Pro^, cannabichromanon (**32**) showed the best fitting within its active centers, followed by cannabinolic acid (**22**) and cannabinol (**21**). However, concerning SARS-CoV-2 PL^pro^, cannabichromanon (**32**), followed by cannabinolic acid (**22**) and cannabicyclolic acid (**41**), revealed the best binding within the active pockets. Furthermore, cannabichromanon (**32**), cannabinolic acid (**22**), and cannabinol (**21**) showed considerable fitting within the active sites of ACE2, as evidenced by their significant ∆G values. This can be interpreted by the formation of multiple bonds between the active moieties of the compounds and the amino acid residues existing at the active sites. It is worth noting that the difference among the compounds in the presence or absence of functional groups, and even the length of the substituted alkyl chains, contribute effectively to the activity of the tested metabolites. This is evidenced by the results displayed in Table 1, where the main difference between the compounds cannabinolic acid (**22**) and cannabinol (**21**) is the existence of an additional carboxylic group that, in turn, increases the activity of compound (**22**) in comparison to compound (**21**). Increasing the length of the alkyl side chain effectively increases the binding affinity between the compounds and the receptor, as evidenced by the higher activity of cannabinol (**21**) compared to compound (**24**); additionally, the latter showed higher activity compared to compound (**23**) with a shorter alkyl side chain. This was also reflected in the enhanced activity of compound (**41**) in comparison to compound (**43**), where the shorter alkyl side chain in the latter decreased the activity to a very great extent; this can be interpreted by fitting to the binding site in terms of the size of the compound relative to that of the active pocket.

The results of ADME/TOPKAT (absorption, distribution, metabolism, excretion, and toxicity) evaluation reflected the considerable pharmacokinetic, pharmacodynamic, and toxicity properties of cannabinol (**21**), cannabinolic acid (**22**), cannabichromanon (**32**), and cannabicyclolic acid (**41**) that showed best fitting scores within the active sites of the tested enzymes. The chemometric analysis served as a simple, rapid technique that made the recognition of the differences and closeness in activity among the examined chemical compounds easier. The data obtained from the binding free energies of the selected cannabinoids versus the three tested enzymes, as well as the values obtained from the ADMET postulation, were subjected to multivariate data analysis which revealed that cannabichromanon and cannabinolic acid showed discriminant activity and hence can be incorporated in pharmaceutical dosage forms to alleviate COVID-19 infection, either alone or in combination with other drugs. 

## 4. Materials and Methods

### 4.1. Selection of Compounds Used in this Study

Forty five compounds previously identified in *Cannabis sativa* L. were selected for this study, as illustrated in Figure 1 and Figure 2. 

### 4.2. Molecular Docking Studies

Molecular modeling for the selected forty five cannabinoids was performed on SARS-CoV-2 M^Pro^ (PDB ID: 6LZE; 1.50 A°), SARS-CoV-2 PL^pro^ (PDB ID: 4OW0; 2.10 A°) and ACE2 (PDB ID: 1R4L; 3.00 A°) employing the C-Docker protocol via Discovery Studio 4.5 (Accelrys Inc., San Diego, CA, USA) as previously discussed [20,21,22,23]. The protein data bank (www.rcsb.org accessed on 22 March 2022) [24] was used to download the X-ray crystal structures of the tested viral enzymes in PDB format. Molecular docking was performed in successive steps. The first step comprised the preparation of the examined protein by eliminating water molecules followed by the addition of hydrogen atoms with subsequent cleaning of the protein structure from the unwanted interaction. CHARMm was used as the forcefield; MMFF94 was employed for the determination of the partial charge and consequently accompanied by minimization of the added hydrogen that was performed in 2000 steps. Previously published data was used to select the binding active sites which had been shown unequivocally to constitute the tested protein catalytic domains. To prepare the tested ligands, the structures of the chosen cannabinoids were primarily drawn in a two-dimensional pattern using ChemDraw 13.0 that was saved in PDB format; then, the default protocol was implemented in Discovery Studio 4.5 which was used to further design the 3D structures of the tested compounds. Molecular docking was performed within the active site of the energy-minimized prepared proteins using the C-Docker protocol. The CHARMm force field was used. However, the binding energy (ΔG) in kcal/mol was calculated by a distance-dependent dielectric implicit solvation model for the best docking poses by adopting the following equation:

Δ*G*_binding_ = E_complex_ − (E_protein_ + E_ligand_)

where

Δ*G*_binding_: the ligand–protein interaction binding energy;

E_complex_: the potential energy for the complex of protein bound with the ligand;

E_protein_: the protein potential energy alone;

E_ligand_: the ligand potential energy alone.

### 4.3. ADME/TOPKAT Evaluation

ADME/TOPKAT (absorption, distribution, metabolism, excretion, and toxicity) evaluation was performed on the chosen cannabinoids by Discovery Studio 4.5 (Accelrys Inc., San Diego, CA, USA). Aqueous solubility, human intestinal absorption, plasma protein binding prediction (PPB), blood-brain barrier penetration (BBB), cytochrome P450 (2D6), as well as hepatotoxicity, were selected as the ADME criteria. The carcinogenic effects on female and male rat FDA, Ames mutagenicity, rat chronic LOAEL, skin and ocular irritant effect, and rat oral LD50 were chosen as toxicity descriptors [25,26].

### 4.4. Chemometric Analysis

Chemometric analysis was performed based upon the values of the binding energies towards different tested enzymes, as well as the values of their pharmacokinetic and pharmacodynamic potential, as obtained from ADMET prediction, aqueous solubility, human intestinal absorption, plasma protein binding prediction (PPB), blood-brain barrier penetration (BBB), cytochrome P450 (2D6), as well as hepatotoxicity. This was performed using principal component analysis (PCA) as an unsupervised pattern recognition technique using CAMO’s Unscrambler^®^ X 10.4 software (Computer-Aided Modeling, As, Norway) [22,27]. 

## 5. Conclusions

*Cannabis sativa* L. is a popular herbaceous plant belonging to the family Cannabinaceae characterized by its richness in cannabinoids. The potential use of forty five cannabinoids previously identified in *C. sativa* to combat COVID-19 infection using in silico studies was discussed. Among the tested compounds, cannabinol, cannabinolic acid, cannabichromanon, and cannabicyclolic acid showed significant activity, evidenced by their best fitting score within the binding sites of three crucial enzymes incorporated in viral replication and host invasion, which were SARS-CoV-2 M^Pro^, SARS-CoV-2 PL^pro^, and ACE2. Most of the tested cannabinoids with significant activity showed promising pharmacokinetic, pharmacodynamic, and toxicity properties, as shown by ADME/TOPAKT prediction, except for cannabidiolic acid cannabigerol, cannabigerolic acid, cannabifuran, and dehydrocannabifuran that showed low absorption lying outside the 99% absorption ellipse. Cannabichromanon and cannabinolic acid showed discriminant activity, as revealed by chemometric analysis. It can be concluded that cannabinol, cannabinolic acid, cannabichromanon, and cannabicyclolic acid could serve as possible candidates to be incorporated into different pharmaceutical dosage forms to alleviate COVID-19 infection; however, further in-depth studies should be conducted to validate the results obtained.

## Figures and Tables

**Table 1:** Binding energies (∆G) (kcal/mol) of the tested cannabinoids using in silico studies within the active centers of SARS-CoV-2 M^Pro^, SARS-CoV-2 PL^pro^ and angiotensin-converting enzyme 2 (ACE2) using pH-ionization mode.

Compound N°	SARS-CoV-2 M^Pro^	SARS-CoV-2 PL^pro^	ACE2
**1**	5.87	8.82	−5.63
**2**	3.80	8.91	−3.20
**3**	16.57	18.76	12.42
**4**	FD	FD	Fd
**5**	5.43	11.35	−0.54
**6**	FD	FD	FD
**7**	−9.86	−10.30	−21.88
**8**	−3.02	2.48	−7.84
**9**	−6.26	−5.98	−12.97
**10**	−2.26	−0.42	−11.52
**11**	−4.93	−1.53	−11.36
**12**	−3.56	4.79	−5.66
**13**	−2.94	−2.22	−9.13
**14**	5.89	9.91	3.45
**15**	−0.36	8.00	−2.83
**16**	−2.26	4.29	−3.51
**17**	3.61	10.54	−1.86
**18**	3.87	6.00	−6.51
**19**	12.51	16.34	3.87
**20**	6.11	14.17	−2.43
**21**	−21.60	−18.60	−30.36
**22**	−23.24	−22.81	−31.34
**23**	−19.40	−15.13	−25.34
**24**	−21.40	−16.33	−27.52
**25**	−16.24	−11.56	−27.85
**26**	FD	FD	FD
**27**	−4.17	−0.21	−7.51
**28**	14.43	15.37	5.34
**29**	15.47	16.11	5.34
**30**	−1.28	2.67	−4.98
**31**	−4.73	−2.41	−9.59
**32**	−33.63	−28.36	−41.77
**33**	2.11	−1.42	−1.97
**34**	−3.44	−2.11	−8.77
**35**	14.62	16.89	3.58
**36**	7.38	11.34	0.08
**37**	10.88	13.37	−2.03
**38**	−18.05	−13.08	−24.23
**39**	−10.05	−6.90	−17.9
**40**	−17.07	−17.41	−22.38
**41**	−19.79	−19.89	−27.15
**42**	−15.12	−9.96	−19.65
**43**	0.78	4.60	−11.60
**44**	9.12	15.58	−1.97
**45**	−19.39	−14.40	−25.61
SARS-CoV-2 M^Pro^ ligand (FHR/PRD_002347)	−4.58	-	-
SARS-CoV-2 PL^pro^ ligand (Y97)	-	−4.05	-
ACE2 ligand (XX5)	-	-	−72.19
Remdesivir	−35.56	2.28	−44.62

FHR/PRD_002347: (~{N}n-[(2~{S})-3-cyclohexyl-1-oxidanylidene-1-[[(2~{S})-1-oxidanylidene-3-[(3~{S})-2-oxidanylidenepyrrolidin-3-yl]propan-2-yl]amino]propan-2-yl]-1~{H}-indole-2-carboxamide). Y97***5***-(azetidin-3-ylamino)-2-methyl-~{N}-[(1~{R})-1-[3-[5-[[[(3~{R})-oxolan-3-yl]amino]methyl]thiophen-2-yl]phenyl]ethyl]benzamide. XX5-(S,S)-2-{1-Carboxy-2-[3-(3,5-dichlorobenzyl)-3H-imidazol-4-yl]-ethylamino}-4-methyl-pentanoic acid. FD: Fail to dock.

**Table 2:** Absorption, distribution, metabolism, excretion, and toxicity (ADMET) properties of the tested cannabinoids using drug discovery software.

Compound	Absorption Level	Solubility Level	BBB Level	PPB Level	CPY2D6	Hepato-Toxic	PSA-2D	Alog p98
**1**	1	2	0	true	Inh	NT	41.631	6.613
**2**	2	2	4	true	NI	NT	79.747	6.243
**3**	0	2	1	true	NI	NT	41.631	4.788
**4**	-	-	-	-	-	-	-	-
**5**	1	2	4	true	NI	Tox	79.747	5.331
**6**	-	-	-	-	-	-	-	-
**7**	0	1	0	true	Inh	Tox	47.046	5.581
**8**	1	1	0	true	NI	Tox	29.745	6.109
**9**	1	2	4	true	NI	Tox	67.861	5.739
**10**	1	2	4	true	NI	Tox	67.861	5.739
**11**	1	1	0	true	NI	Tox	29.745	6.109
**12**	0	1	0	true	NI	Tox	29.745	5.653
**13**	1	2	4	true	NI	Tox	67.861	5.739
**14**	0	2	0	true	NI	Tox	29.745	4.284
**15**	0	2	0	true	NI	Tox	29.745	5.197
**16**	0	2	1	true	NI	Tox	67.861	4.827
**17**	3	2	4	true	Inh	NT	41.631	7.34
**18**	2	2	4	true	NI	NT	79.747	6.969
**19**	1	2	0	true	Inh	NT	41.631	6.427
**20**	1	2	0	true	Inh	NT	41.631	6.427
**21**	2	2	4	true	NI	NT	79.747	6.057
**22**	1	1	0	true	NI	Tox	29.745	6.223
**23**	1	1	4	true	NI	Tox	67.861	5.853
**24**	0	1	0	true	NI	Tox	29.745	5.767
**25**	1	2	4	true	NI	Tox	41.631	6.659
**26**	-	-	-	-	-	-	-	-
**27**	1	3	0	true	NI	true	29.745	6.109
**28**	1	3	0	true	NI	NT	52.954	5.791
**29**	1	3	4	false	NI	NT	67.861	5.614
**30**	0	1	0	true	NI	NT	29.745	6.58
**31**	1	2	4	true	NI	NT	67.861	6.21
**32**	1	2	1	true	NI	NT	64.347	4.336
**33**	0	2	0	true	NI	Tox	29.745	5.668
**34**	0	2	1	true	NI	NT	67.861	5.297
**35**	0	2	1	true	NI	NT	50.561	5.264
**36**	0	2	4	true	NI	Tox	88.677	4.893
**37**	1	2	4	true	NI	Tox	88.677	4.893
**38**	3	1	4	true	NI	Tox	33.369	7.082
**39**	2	1	4	true	NI	Tox	33.369	6.886
**40**	0	1	0	true	NI	Tox	29.745	5.601
**41**	0	2	1	true	NI	Tox	67.861	5.23
**42**	0	2	0	true	NI	Tox	29.745	4.688
**43**	0	2	1	true	NI	Tox	71.376	4.256
**44**	0	2	0	true	NI	Tox	41.631	5.701
**45**	0	2	1	true	NI	NT	71.376	4.176

0, 1, 2, and 3 indicates good, moderate, low and very low absorption, respectively; 0, 1, 2, 3, 4, and 5 indicates extremely low, very low but possible, low, good, optimal, and too soluble, respectively; 0, 1, 2, 3, and 4 denote very high, high, medium, low, and undefined, penetration via BBB respectively. PBB: plasma protein binding; false = less than 90%, true = more than 90%; Inh: Inhibitor; NI: non-inhibitor; NT: non-toxic; Tox: toxic.

**Table 3:** TOPKAT prediction of the tested cannabinoids using drug discovery software.

Compound	Ames Prediction	Rat Oral LD50	Rat Chronic LOAEL	Skin Irritancy	Ocular Irritancy	Rat Female FDA	Rat Male FDA
**1**	Non-Mutagen	0.75	0.21	Moderate	None	Non-Carcinogen	Non-Carcinogen
**2**	Non-Mutagen	1.38	0.21	None	Severe	Non-Carcinogen	Non-Carcinogen
**3**	Non-Mutagen	0.59	0.11	Moderate	Severe	Non-Carcinogen	Non-Carcinogen
**4**	-	-	-	-	-	-	-
**5**	Non-Mutagen	1.17	0.16	None	Severe	Non-Carcinogen	Non-Carcinogen
**6**	-	-	-	-	-	-	-
**7**	Non-Mutagen	1.52	0.09	Moderate	Severe	Non-Carcinogen	Non-Carcinogen
**8**	Non-Mutagen	0.64	0.05	Moderate	Severe	Non-Carcinogen	Non-Carcinogen
**9**	Non-Mutagen	0.96	0.03	None	Severe	Non-Carcinogen	Non-Carcinogen
**10**	Non-Mutagen	0.59	0.04	None	Severe	Non-Carcinogen	Non-Carcinogen
**11**	Non-Mutagen	0.61	0.04	Moderate	Severe	Non-Carcinogen	Non-Carcinogen
**12**	Non-Mutagen	0.76	0.03	Moderate	Severe	Non-Carcinogen	Non-Carcinogen
**13**	Non-Mutagen	1.01	0.04	None	Severe	Non-Carcinogen	Non-Carcinogen
**14**	Non-Mutagen	0.43	0.02	Moderate	Severe	Non-Carcinogen	Non-Carcinogen
**15**	Non-Mutagen	0.46	0.02	Moderate	Severe	Non-Carcinogen	Non-Carcinogen
**16**	Non-Mutagen	0.73	0.02	None	Severe	Non-Carcinogen	Non-Carcinogen
**17**	Non-Mutagen	2.26	0.30	Moderate	None	Non-Carcinogen	Non-Carcinogen
**18**	Non-Mutagen	4.14	0.29	None	None	Non-Carcinogen	Non-Carcinogen
**19**	Non-Mutagen					Non-Carcinogen	Non-Carcinogen
**20**	Non-Mutagen	1.89	0.23	Moderate	None	Non-Carcinogen	Non-Carcinogen
**21**	Non-Mutagen	3.52	0.22	None	None	Non-Carcinogen	Non-Carcinogen
**22**	Non-Mutagen	2.31	0.23	None	Severe	Non-Carcinogen	Non-Carcinogen
**23**	Non-Mutagen	3.63	0.18	None	Mild	Non-Carcinogen	Non-Carcinogen
**24**	Non-Mutagen	2.57	0.11	None	Mild	Non-Carcinogen	Non-Carcinogen
**25**	Non-Mutagen	3.90	0.90	None	Mild	Non-Carcinogen	Non-Carcinogen
**26**	-	-	-	-	-	-	-
**27**	Non-Mutagen	0.65	0.05	Moderate	Severe	Non-Carcinogen	Non-Carcinogen
**28**	Non-Mutagen	5.01	0.42	Mild	None	Non-Carcinogen	Non-Carcinogen
**29**	Non-Mutagen	5.32	0.13	Mild	None	Non-Carcinogen	Non-Carcinogen
**30**	Non-Mutagen	1.85	0.07	Moderate	Severe	Non-Carcinogen	Non-Carcinogen
**31**	Non-Mutagen	2.94	0.06	None	Mild	Non-Carcinogen	Non-Carcinogen
**32**	Non-Mutagen	3.64	0.22	None	Moderate	Non-Carcinogen	Non-Carcinogen
**33**	Non-Mutagen	1.33	0.04	Moderate	Severe	Single-Carcinogen	Non-Carcinogen
**34**	Non-Mutagen	2.13	0.03	None	Mild	Non-Carcinogen	Non-Carcinogen
**35**	Non-Mutagen	1.85	0.04	Moderate	Severe	Non-Carcinogen	Non-Carcinogen
**36**	Non-Mutagen	2.3	0.03	None	Severe	Non-Carcinogen	Non-Carcinogen
**37**	Non-Mutagen	1.34	0.03	None	Severe	Non-Carcinogen	Non-Carcinogen
**38**	Non-Mutagen	0.09	0.23	Severe	Severe	Non-Carcinogen	Non-Carcinogen
**39**	Non-Mutagen	0.34	0.24	None	Severe	Non-Carcinogen	Non-Carcinogen
**40**	Non-Mutagen	0.98	0.07	Moderate	Severe	Non-Carcinogen	Non-Carcinogen
**41**	Non-Mutagen	1.54	0.06	None	Severe	Single-Carcinogen	Non-Carcinogen
**42**	Non-Mutagen	0.71	0.04	None	Severe	Single-Carcinogen	Non-Carcinogen
**43**	Non-Mutagen	3.24	0.05	None	Severe	Non-Carcinogen	Non-Carcinogen
**44**	Non-Mutagen	0.63	0.16	Moderate	None	Non-Carcinogen	Non-Carcinogen
**45**	Non-Mutagen	1.15	0.09	None	Severe	Non-Carcinogen	Non-Carcinogen

Both rat oral LD50 and rat chronic LOAEL are expressed in g/kg body weight.

## Data Availability

Data are available in the manuscript.

## Supplementary Materials

The following supporting information can be downloaded at: https://www.mdpi.com/article/10.3390/molecules27092797/s1, Figure S1: 2D and 3D binding of cannabinolic acid (A) and cannabinol (B) within the active sites of SARS-CoV-2 M^Pro^; Figure S2: 2D and 3D binding of cannabinolic acid (A) and cannabicyclolic acid (B) within the active sites of SARS-CoV-2 PL^pro^; Figure S3: 2D and 3D binding of cannabinolic acid (A) and cannabinol (B) within the active sites of ACE-2; Figure S4: The presence of cannabichromanon (32) inside the binding pockets of SARS-CoV-2 M^Pro^ (A), SARS-CoV-2 PL^pro^ (B) and ACE-2 (C) displaying areas of aromaticity, hydrogen bond formation, hydrophobicity and solvent accessibility (SAS) of the receptor residues; Figure S5: 2D binding modes of the respective co-crystalized ligands in SARS-CoV-2 M^Pro^ (A), SARS-CoV-2 PL^pro^ (B) and ACE2 (C); Figure S6: 2D binding modes of remdesivir in SARS-CoV-2 M^Pro^ (A), SARS-CoV-2 PL^pro^ (B) and ACE2 (C).
