# Supplementary material for: The Role of Cannabis sativa L. as a Source of Cannabinoids against Coronavirus 2 (SARS-CoV-2): An In Silico Study to Evaluate Their Activities and ADMET Properties
Source: Molecules. 2022 Apr 27;27(9):2797. doi: 10.3390/molecules27092797 (PMC9104455; doi:10.3390/molecules27092797)
Supplement: Supplementary file 1 [file molecules-27-02797-s001.zip › molecules-1671131-supplementary.pdf]

**Supplementary material**

# The role of *Cannabis sativa* L as a Source of Cannabinoids against Coronavirus 2 (SARS-CoV-2): An *in Silico* Study to Evaluate their Activities and ADMET Properties

**Figure S1** 2D and 3D binding of cannabinolic acid **(A)** and cannabinol **(B)** within the active sites of SARS-CoV-2 $M^{Pro}$

**Figure S2** 2D and 3D binding of cannabinolic acid **(A)** and cannabicyclolic acid **(B)** within the active sites of SARS-CoV- $2PL^{pro}$

**Figure S3** 2D and 3D binding of cannabinolic acid **(A)** and cannabinol **(B)** within the active sites of ACE-2

**Figure S4** The presence cannabichromanon **(32)** inside the binding pockets of SARS-CoV-2 $M^{Pro}$ (**A**), SARS-CoV-2 $PL^{pro}$ (**B**) and ACE-2 (**C**) displaying areas of aromaticity, hydrogen bond formation, hydrophobicity and solvent accessibility (SAS) of the receptor residues

**Figure S5**2D binding modes of the respective co-crystalized ligands in SARS-CoV-2 $M^{Pro}$ (A), SARS-CoV-2 $PL^{pro}$ (B) and ACE2 (C)

**Figure S6**2D binding modes of remdesivir in SARS-CoV-2 $M^{Pro}$ (A), SARS-CoV-2 $PL^{pro}$ (B) and ACE2 (C)

**Figure S1** 2D and 3D binding of cannabinolic acid **(A)** and cannabinol **(B)** within the active sites of SARS-CoV-2 $M^{Pro}$

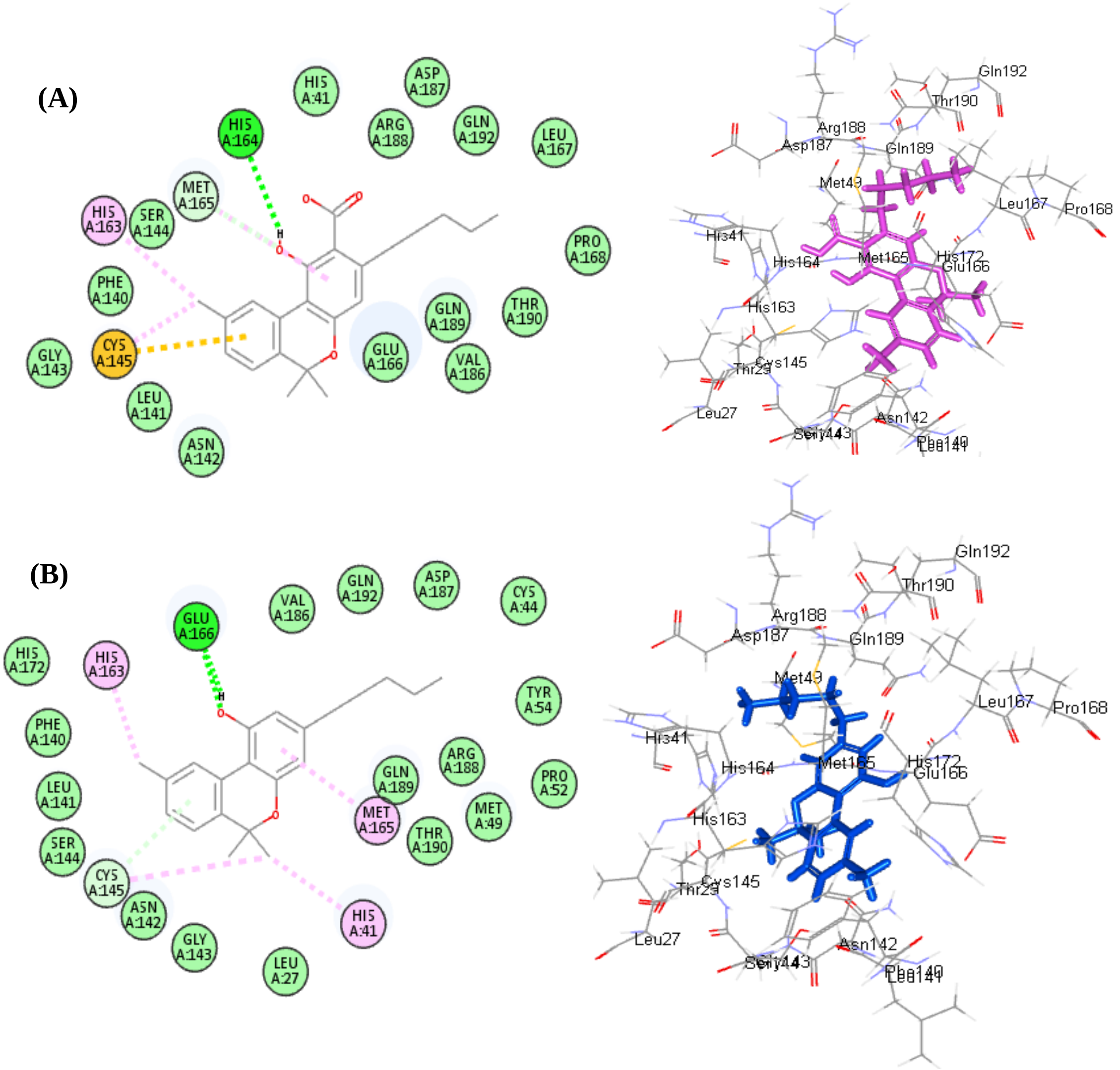

**Figure S2** 2D and 3D binding of cannabinolic acid **(A)** and cannabicyclolic acid **(B)** within the active sites of , SARS-CoV- 2PL$^{pro}$

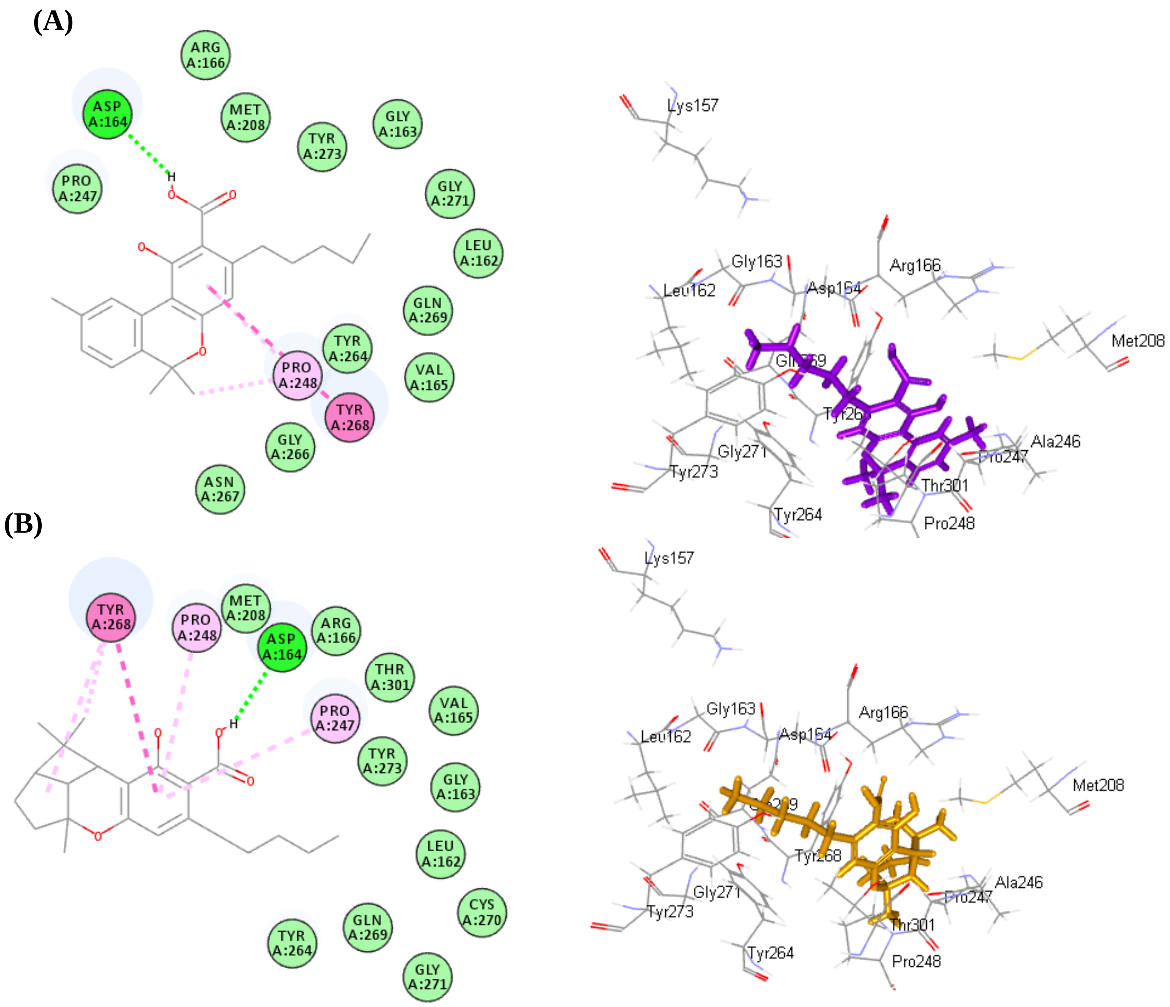

**Figure S3** 2D and 3D binding of cannabinolic acid **(A)** and cannabinol **(B)** within the active sites of ACE-2

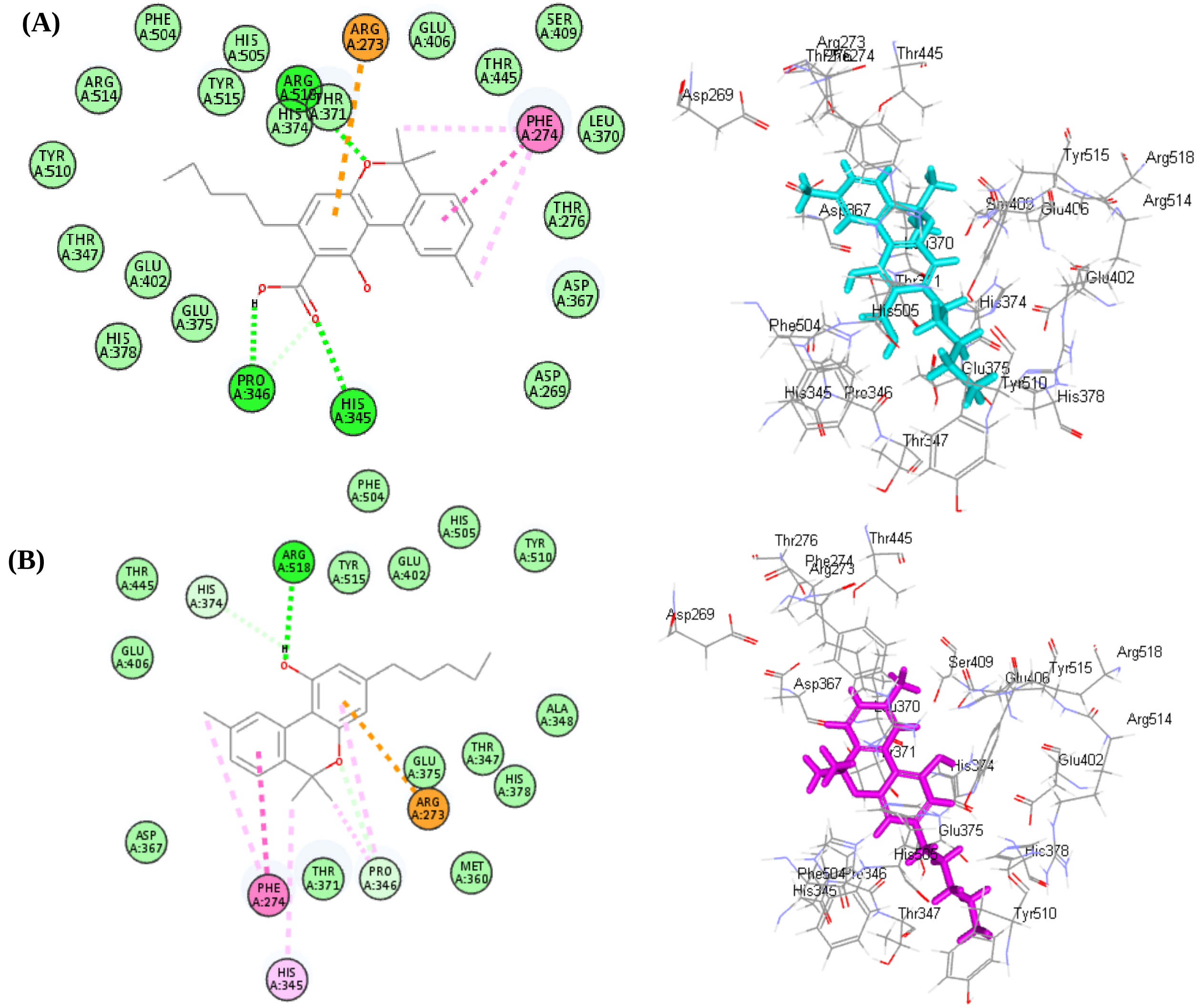

**Figure S4** The presence cannabichromanon **(32)** inside the binding pockets of SARS-CoV-2 M$^{Pro}$ (**A**), SARS-CoV-2 PL$^{pro}$ (**B**) and ACE-2 (**C**) displaying areas of aromaticity, hydrogen bond formation, hydrophobicity and solvent accessibility (SAS) of the receptor residues

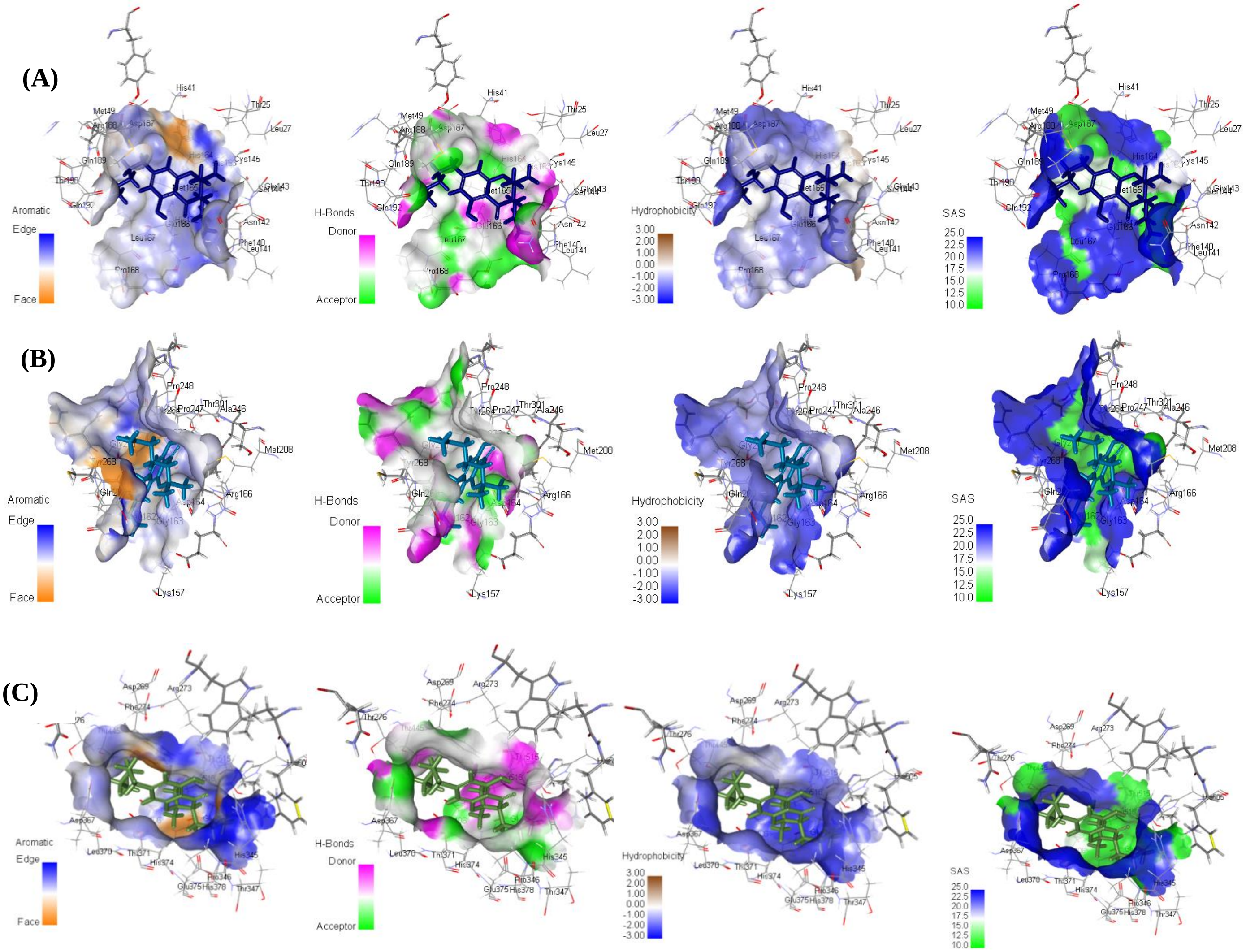

**Figure S5**2D binding modes of the respective co-crystalized ligands in SARS-CoV-2 M[Pro] **(A),** SARS-CoV-2 PL[pro] **(B)** and ACE2 **(C)**

**(A)**

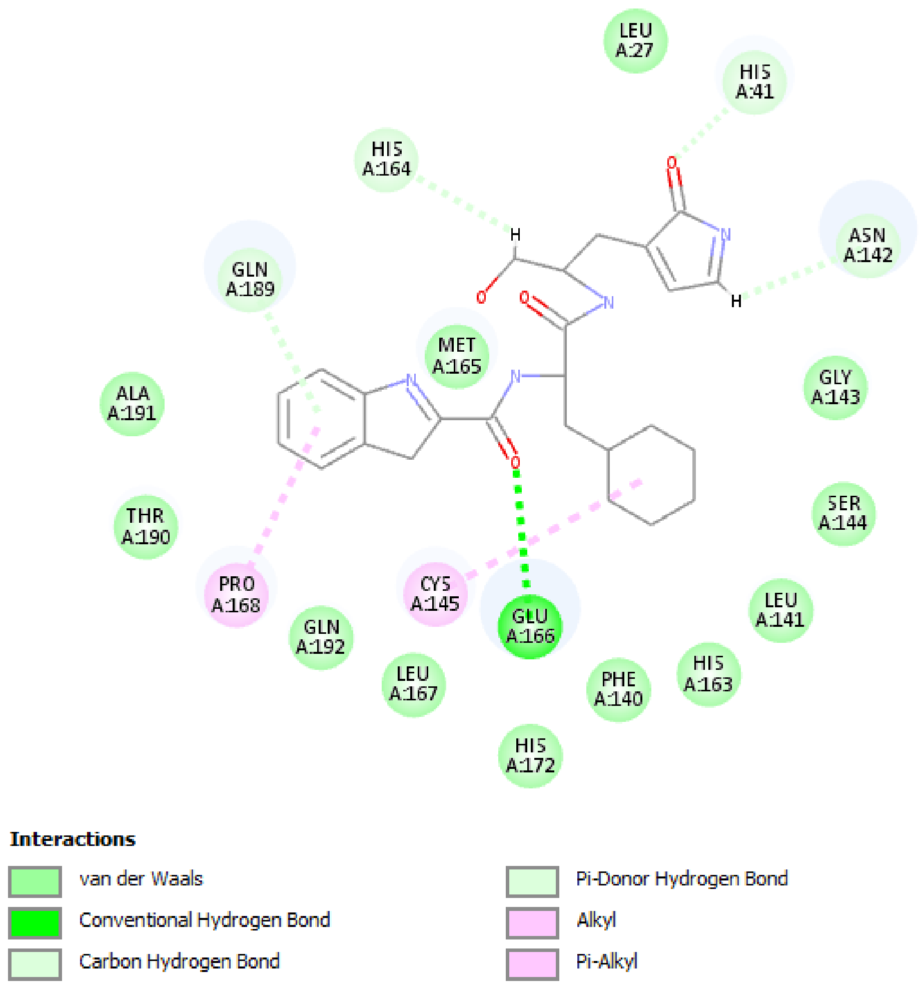

(B)

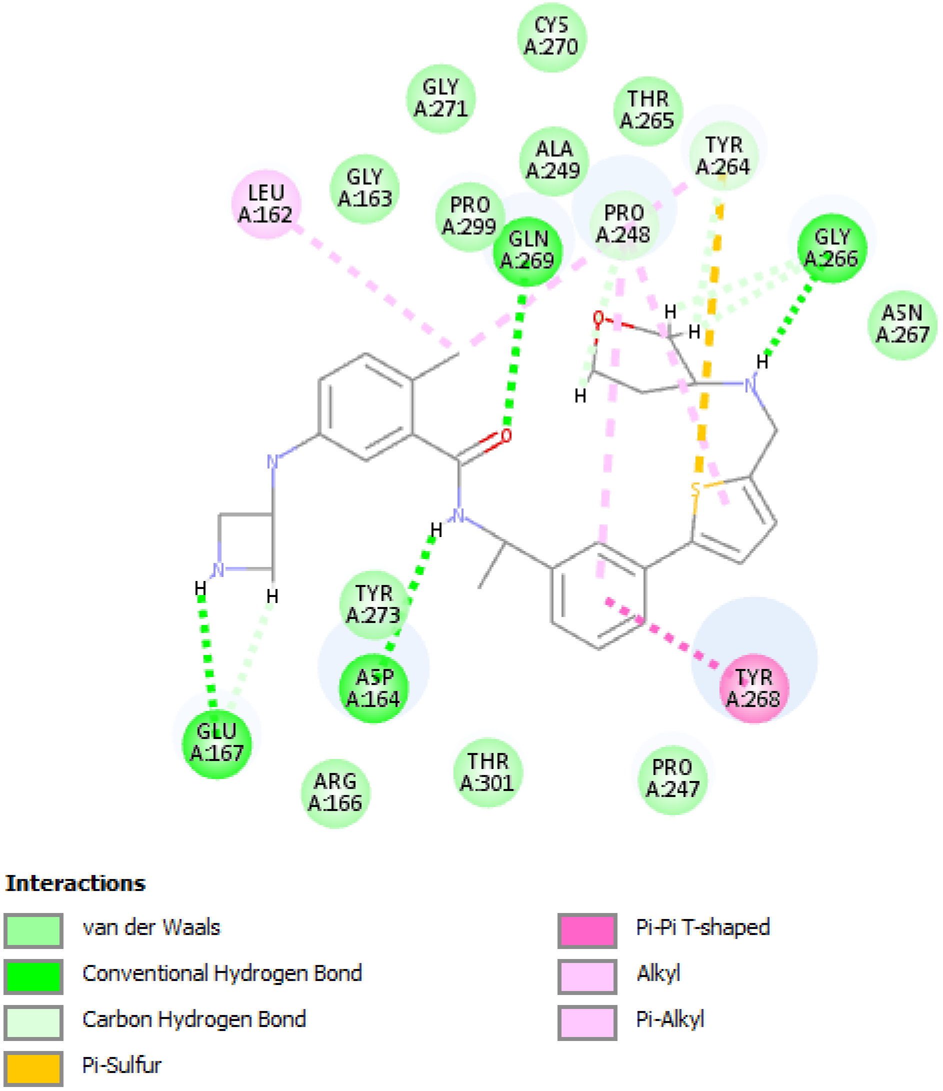
CYS A:270
GLY A:271
THR A:265
TYR A:264
ALA A:249
GLY A:163
LEU A:162
PRO A:299
PRO A:248
GLN A:269
GLY A:266
ASN A:267
TYR A:273
ASP A:164
TYR A:268
GLU A:167
ARG A:166
THR A:301
PRO A:247
Interactions
van der Waals
Conventional Hydrogen Bond
Carbon Hydrogen Bond
Pi-Sulfur
Pi-Pi T-shaped
Alkyl
Pi-Alkyl

**(C)**

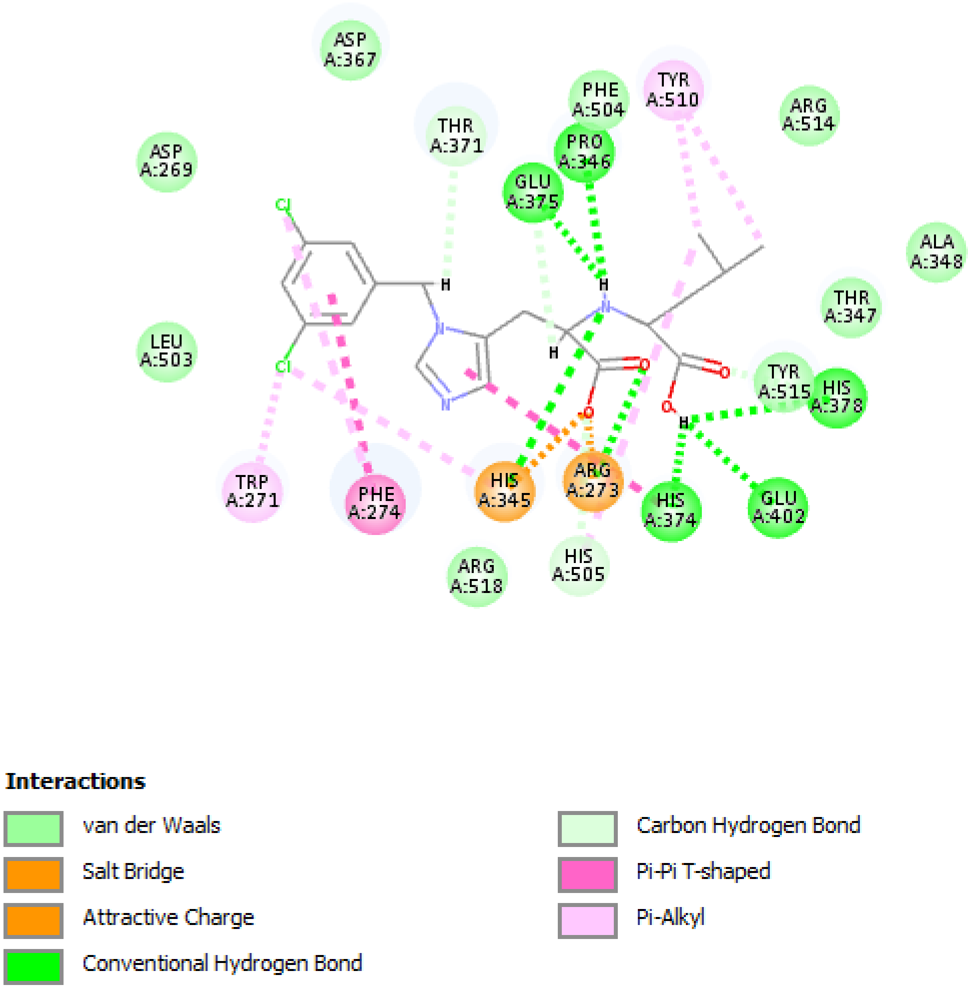
ASP A:367
THR A:371
PHE A:504
TYR A:510
ARG A:514
ASP A:269
PRO A:346
GLU A:375
ALA A:348
THR A:347
LEU A:503
TYR A:515
HIS A:378
TRP A:271
PHE A:274
HIS A:345
ARG A:273
HIS A:374
GLU A:402
ARG A:518
HIS A:505
Cl
Cl
N
N
N
H
H
H
H
O
O
O
O
Interactions
van der Waals
Salt Bridge
Attractive Charge
Conventional Hydrogen Bond
Carbon Hydrogen Bond
Pi-Pi T-shaped
Pi-Alkyl

**Figure S6**2D binding modes of remdesivir in SARS-CoV-2 M$^{Pro}$ **(A),** SARS-CoV-2 PL$^{pro}$ **(B)** and ACE2 **(C)**

**(A)**

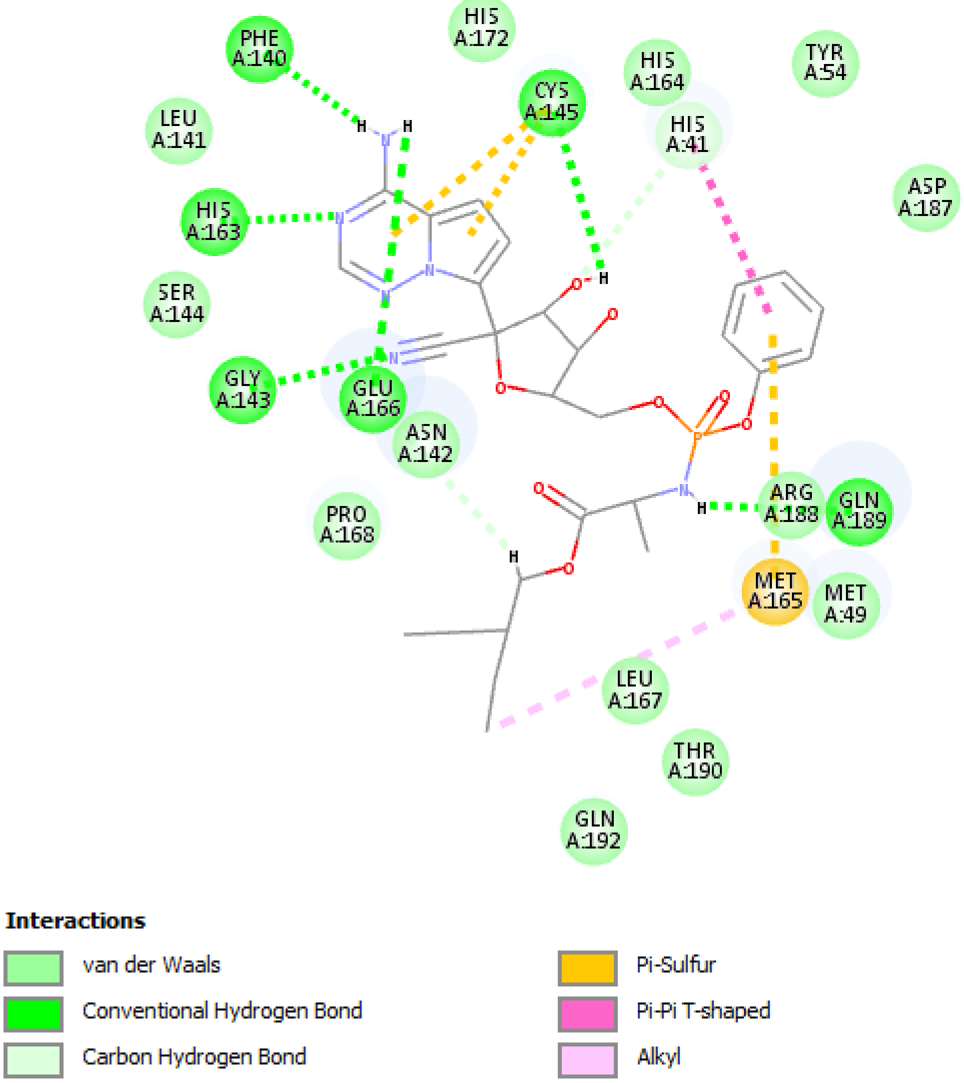

**(B)**

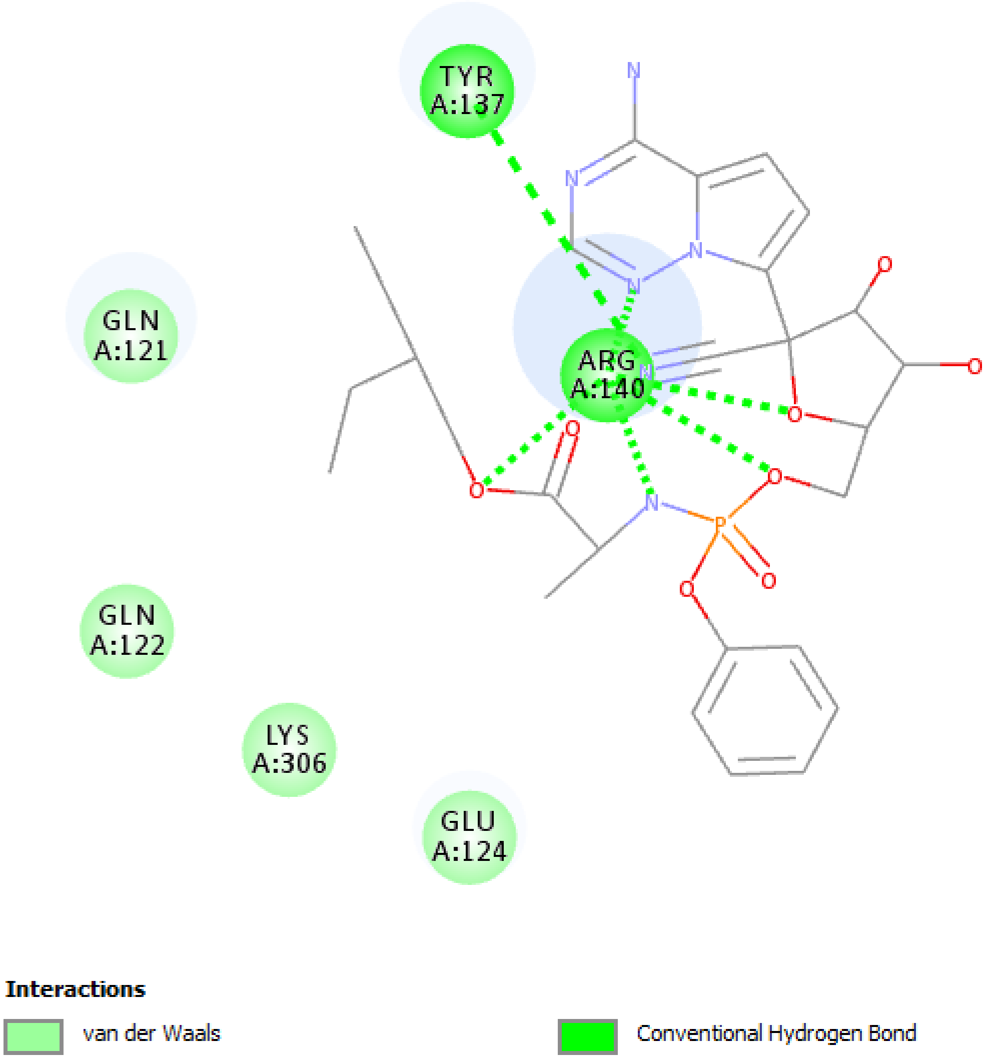

TYR A:137
GLN A:121
ARG A:140
GLN A:122
LYS A:306
GLU A:124
Interactions
van der Waals
Conventional Hydrogen Bond

(C)

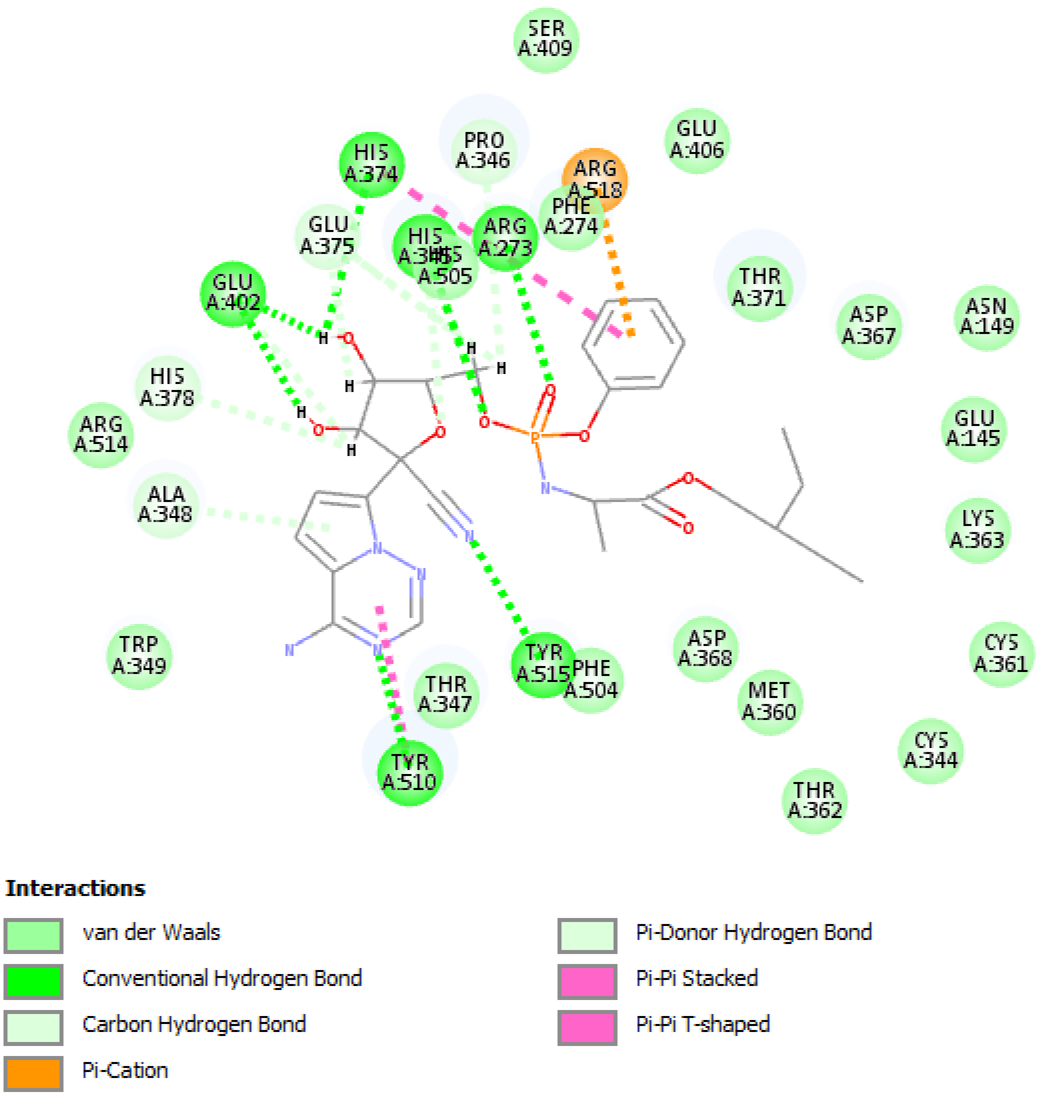
SER A:409
PRO A:346
GLU A:406
HIS A:374
ARG A:518
PHE A:274
GLU A:375
ARG A:273
A:505
GLU A:402
THR A:371
ASP A:367
ASN A:149
HIS A:378
ARG A:514
GLU A:145
ALA A:348
LYS A:363
TRP A:349
TYR A:515
PHE A:504
ASP A:368
CYS A:361
THR A:347
MET A:360
CYS A:344
TYR A:510
THR A:362
Interactions
van der Waals
Conventional Hydrogen Bond
Carbon Hydrogen Bond
Pi-Cation
Pi-Donor Hydrogen Bond
Pi-Pi Stacked
Pi-Pi T-shaped
